# Whole Exome Sequencing Identified Novel *ARMC9* Variations in Two Cases With Joubert Syndrome

**DOI:** 10.3389/fgene.2022.817153

**Published:** 2022-02-04

**Authors:** Hao Wang, Guanjun Luo, Wensheng Hu, Jin Mei, Yue Shen, Min Wang, Yuan Tan, Yang Yang, Chao Lu, Yong Zhao, Ming Qi

**Affiliations:** ^1^ Department of Cell Biology and Medical Genetics, School of Medicine, Zhejiang University, Hangzhou, China; ^2^ Prenatal Diagnosis Center, Hangzhou Women’s Hospital, Hangzhou, China; ^3^ Child Rehabilitation Department, Nanhai Affiliated Maternity and Children’s Hospital of Guangzhou University of Chinese Medicine, Foshan, China; ^4^ Department of Obstetrics and Gynecology, Hangzhou Women’s Hospital Hangzhou, Hangzhou, China; ^5^ National Research Institute for Family Planning, Beijing, China

**Keywords:** Joubert syndrome, ARMC9 gene, whole-exome sequencing, molecular dynamic analysis, minigene system

## Abstract

**Background:** Biallelic variations in the armadillo repeat-containing 9 (*ARMC9*) gene were recently defined to cause Joubert syndrome (JS) type thirty. In this study, two unrelated families with probands displaying typical indications of JS were enrolled and underwent a series of clinical and genetic investigations.

**Methods:** Routine evaluation including magnetic resonance imaging (MRI) was carried out. Whole-exome sequencing (WES) was performed on the probands to detect causative variants. Next, *in silico* structural and molecular dynamic (MD) analysis was conducted on the missense variant for analyzing its intramolecular impact. Meanwhile, an *in vitro* study with the minigene system was performed to explore the specific impact on mRNA splicing of another variant.

**Results:** Two unrelated patients from two different families came to our hospital exhibiting typical JS presentations, such as the “molar tooth sign.” Using WES, we identified that both probands carried the compound heterogeneous variants in *ARMC9* (NM_025139.6), with c.1878+1G > A and c.895C > T (p.Arg299Ter) in family 1 and c.1878+1G > A and c.1027C > T (p.Arg343Cys) in family 2. These variants were inherited from their unaffected parents by Sanger sequencing, respectively, and *ARMC9* c.895C > T (p.Arg299Ter) and c.1878+1G > A were novel variants. *In silico* analysis indicated the c.1027C > T (p.Arg343Cys) would likely affect the secondary structure of the ARMC9 protein. The minigene study demonstrated that the splice site variant c.1878+1G > A abolished the canonical donor site, resulting in an 18bp intronic retention of intron 20.

**Conclusion:** The findings in this study expanded the mutation spectrum of *ARMC9*-associated JS, and we suggested that the function of ARMC9 in the pathogenesis of JS might involve the development of primary cilia, after discussing the function of the ARMC9 protein.

## Introduction

Joubert syndrome (JS, MIM #213300), a spectrum of rare congenital disorders, was first described by Marie Joubert in 1969 (Joubert et al., 1969) and mainly characterized by hypotonia, intellectual disability/developmental delay, and the distinctive cerebellar and brain stem malformation called the molar tooth sign (MTS) (Brancati et al., 2010). Other additional features consist of oculomotor apraxia and abnormal respiratory pattern (Parisi, 2019). In general, the breathing abnormalities improve with age, truncal ataxia develops over time, and acquisition of gross motor milestones is delayed; cognitive abilities are variable, ranging from severe intellectual disability to normal (Parisi et al., 2003; Brancati et al., 2010). Some JS patients may have multisystem organ involvement including retinal dystrophy, renal disease, ocular colobomas, occipital encephalocele, hepatic fibrosis, polydactyly, oral hamartomas, and endocrine abnormalities (Parisi et al., 2003; Vilboux et al., 2017). The term “Joubert syndrome and related disorders” (JSRD) was extensively used in recent years because of the strong clinical heterogeneity of these conditions (Brancati et al., 2010).

The estimated incidence of JS ranges between 1/80,000 and 1/100,000 in live births (Brancati et al., 2010). Till date, pathogenic variants in more than 35 genes are known to cause JS. The vast majority of these genes conform to the autosomal recessive pattern, and one (*OFD1*) is X-linked (Parisi et al., 2003; Van De Weghe et al., 2017). All of the gene products localize in and around the primary cilium, rendering JS a canonical ciliopathy (Van De Weghe et al., 2017). A molecular diagnosis of JS can be established in about 62–97% of individuals with clinical manifestations of JS by analyzing these genes, depending on the specific study (Parisi et al., 2003; Bachmann-Gagescu et al., 2015; Shaheen et al., 2016). The *ARMC9* gene (armadillo repeat-containing protein 9, MIM *617612), which encodes a basal body protein, is a recently defined causative gene for JS type thirty (Van De Weghe et al., 2017). Van De Weghe et al*.* initially identified deleterious *ARMC9* variants in 8 JS families, accounting for approximately 1% of JS pathogenesis in their cohort (Van De Weghe et al., 2017). Subsequently, reports by Kar et al*.* and Latour et al*.* strengthened this pathogenic relationship and provided new functional experimental clues (Kar et al., 2018; Latour et al., 2020). However, since only a few causative variants were identified, a fine genotype–phenotype association of *ARMC9*-causing JS is still to be established to better understand this condition.

Here, in the present study, we recruited two families with probands displaying typical JS manifestations. These probands are unrelated. A comprehensive clinical evaluation and genetic detection were conducted to provide detailed characterization of genotype and phenotype. Biallelic variants in the *ARMC9* gene were identified in both probands, involving two novel variants. Bioinformatic and *in vitro* experimental validations were performed to confirm the pathogenicity of these variants.

## Material and Methods

The present study was approved by the Ethics Committee of Hangzhou Women’s Hospital [No. (1)-04-K-2022]. Informed consent was provided by all the participants or their statutory guardians included in this study. All procedures performed in the present study were in accordance with the Declaration of Helsinki 1964 and its later amendments or comparable ethical standards.

### Subjects

We recruited two families with probands displaying developmental delay and performed a thorough clinical evaluation on them. Magnetic resonance imaging (MRI) was conducted on the patients to reveal abnormalities in the central nervous system (CNS).

### Exome and Sanger Sequencing

Genomic DNA was extracted from the peripheral blood sample from each of the probands and their parents using the QIAamp DNA Blood Mini Kit (Qiagen, Germany).

Whole-exome sequencing (WES) was employed to detect the sequence variants in the probands’ samples, as described in a previous study (Huang et al., 2021). Briefly, the target-region sequence enrichment was performed using the Agilent Sure Select Human Exon Sequence Capture Kit (Agilent, United States). DNA libraries were tested by quantitative PCR, where the size, distribution, and concentration were determined using Agilent Bioanalyzer 2100 (Agilent, United States). Along with ∼150 bp pair-end reads, the NovaSeq6000 platform (Illumina, Inc.) was used for sequencing of DNA with ∼300 pM per sample with the NovaSeq Reagent Kit. Sequencing raw reads (quality level Q30% > 90%, criteria referring to https://www.illumina.com/science/technology/next-generation-sequencing/plan-experiments/quality-scores.html) were aligned to the human reference genome (accession No. hg19/GRCh37) using the Burrows Wheeler Aligner tool, and PCR duplicates were removed using Picardv1.57. Variant calling was performed with the Verita Trekker^®^ Variants Detection System (v2.0; Berry Genomics, China) and Genome Analysis Tool Kit (https://software.broadinstitute.org/gatk/). Then, variants were annotated and interpreted using ANNOVAR (v2.0) (Wang et al., 2010) and Enliven^®^ Variants Annotation Interpretation systems (Berry Genomics), based on the common guidelines by the American College of Medical Genetics and Genomics (ACMG) (Richards et al., 2015). To assist in the interpretation of variant pathogenicity, we referred to the allele frequency from gnomAD exome databases (http://gnomad.broadinstitute.org) and HGMD (Human Gene Mutation Database) pro v2019; Revel score (a combined method of pathogenicity prediction) (Ioannidis et al., 2016) and pLI score (representing the tolerance for truncating variants) were also employed.

Sanger sequencing was performed as a validation method with the 3500DX Genetic Analyzer (Applied Biosystems, United States). The PCR primers for sequencing were included in Supplementary Material S1.

### Conservational, Structural, and Molecular Dynamic Analysis

The evolutionary conservation of the affected amino acid (AA) residue by missense variant was analyzed using the MEGA7 online tool (http://www.megasoftware.net/previousVersions.php) with default parameters. The SWISS-MODEL program was applied to model the segmental ARMC9-LisH domain (AA 321–570) containing the missense variant site with the AF-Q7Z3E5-F1 template (https://alphafold.ebi.ac.uk/entry/Q7Z3E5).

The molecular dynamic (MD) analysis was generated by GROMACS (version 2020.6) (Rakhshani et al., 2019). A 120ns MD simulation was carried out on the wild-type (WT) and p. Arg343Cys mutant ARMC9 models. The CHARMM36 force field was applied to add hydrogen atoms and N-terminal and C-terminal patches to the models (Gutiérrez et al., 2016). The wild-type or the mutant structure of the protein was immersed in cubic boxes which contain water and placed at least 1.0 nm from the box edge. Na^+^ and Cl^−^ ions were used for neutralization. MD simulation was performed at a temperature of 300 K for 120 ns after energy minimization and equilibration. The following GROMACS distribution programs were used in MD trajectories: gmxrms, gmxrmsf, gmx gyrate, gmxsasa, gmxhbond, and gmxdssp. These MD analyses generated parameter values for root-mean-square deviation (RMSD), root-mean-square fluctuation (RMSF), radius of gyration (Rg), solvent accessible surface area (SASA), number of h-bonds, and secondary structure (SS).

### *In Vitro* Experimental Validation

To investigate the impact of the c.1878+1G > A variant on mRNA splicing, an *in vitro* validation experiment was conducted.

Briefly, the *ARMC9* wild-type (WT) and *ARMC9*: c.1878+1G > A mutant minigene plasmids were constructed with the in-house designed pMini-CopGF expression backbone (DNA sequences and plasmid backbone details were included in Supplementary Material S2). Subsequently, HEK (human embryonic kidney) 293T cells were transfected by these plasmids, and the RNA sample was extracted and reversely transcripted into cDNA. The particular impact on mRNA splicing was analyzed *via* PCR fragment amplification and Sanger sequencing (details in Supplementary Material S2).

## Results

### Clinical Manifestation

**FIGURE 1:**
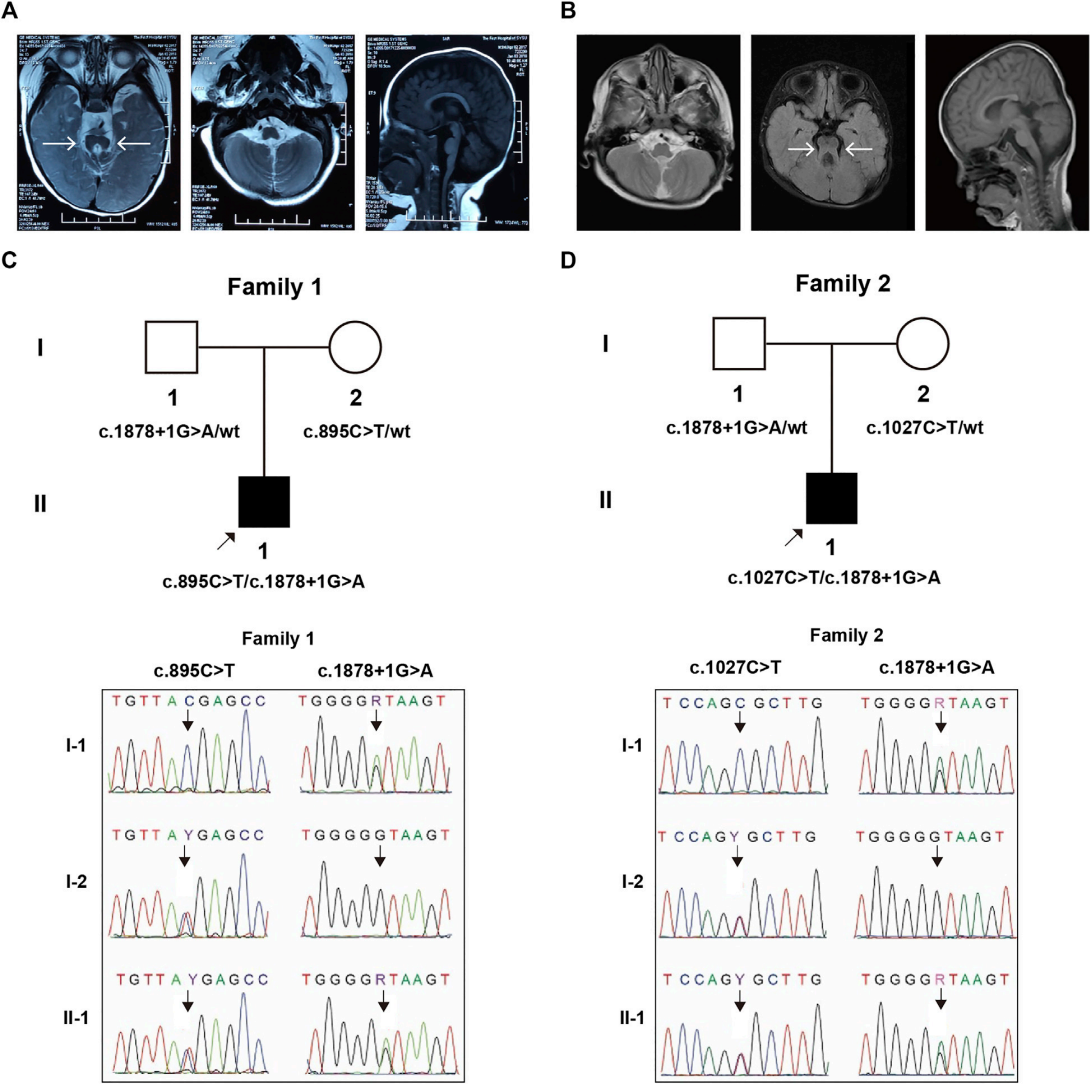
MRI imaging and genetic segregation of variants within trios. The isthmus is abnormally thickened and lengthened, forming a molar-like structure between the superior cerebellar peduncle and the dysplastic cerebellum vermis in Patient 1 **(A)** and Patient 2 **(B)**. The segregation confirmation in family 1 **(C)** and family 2 **(D)**.

Patient 1 (II-1, Figure 1C) was a 16-month-old boy with development delay, ptosis, and abnormal breathing pattern, whose parents had previously terminated a pregnancy due to trisomy 18. They are non-consanguineous and had no history of congenital anomalies or recurrent miscarriages. Apgar scores of the proband were 10 at 1 minute, the birth weight was 2,650 g, and the length was 46 cm. He had delayed crying at birth and feeding difficulties in the neonatal period. Episodic tachypnea and apnea were obvious, especially at sleeping time, but were gradually relieved during infancy. He was referred to the hospital for developmental delay at the age of 4 months. Large head with frontal bossing, left ptosis, depressed nasal bone, and low-set ears were noticed, along with hypotonia and ocular motor apraxia. He could not lift his neck nor support the body with the elbow. JS diagnosis was made based on the brain MRI findings of the absence of inferior vermis of the cerebellar and the molar tooth sign (Figure 1A). Other evaluations including kidney and liver functional tests, ultrasonography of abdominal, funduscopy, ERG, chromosomal karyotype, and array CGH were normal.

Patient 2 (II-1, Figure 1D) was a 5-year-old boy with poor motor and language ability, abnormal skull and teeth, ptosis, hypotonia, unsteady walking, and occasional apnea. His parents came from non-consanguineous Han families and did not have any significant family history. He was referred to the hospital because of developmental delay at 3 months. He could not look up until he was 1.5 years old, sit up until he was 2 years old, and walk independently until he was 5 years old. JS diagnosis was made based on cerebral MRI findings (molar tooth sign, Figure 1B). Karyotype and SNP-array results were normal.

### Molecular and Genetic Analysis

WES identified a compound heterozygous variation in the *ARMC9* (NM_025139.6) gene consisting of two variants, c.1878+1G > A and c.895C > T (p.Arg299Ter) in Patient 1, which were inherited from each parent, respectively (Figure 1C). Similarly, WES also identified a compound heterozygous variation consisting of two variants, *ARMC9*: c.1878+1G > A and c.1027C > T (p.Arg343Cys), inherited from each of his parents, respectively (Figure 1D).

**FIGURE 2:**
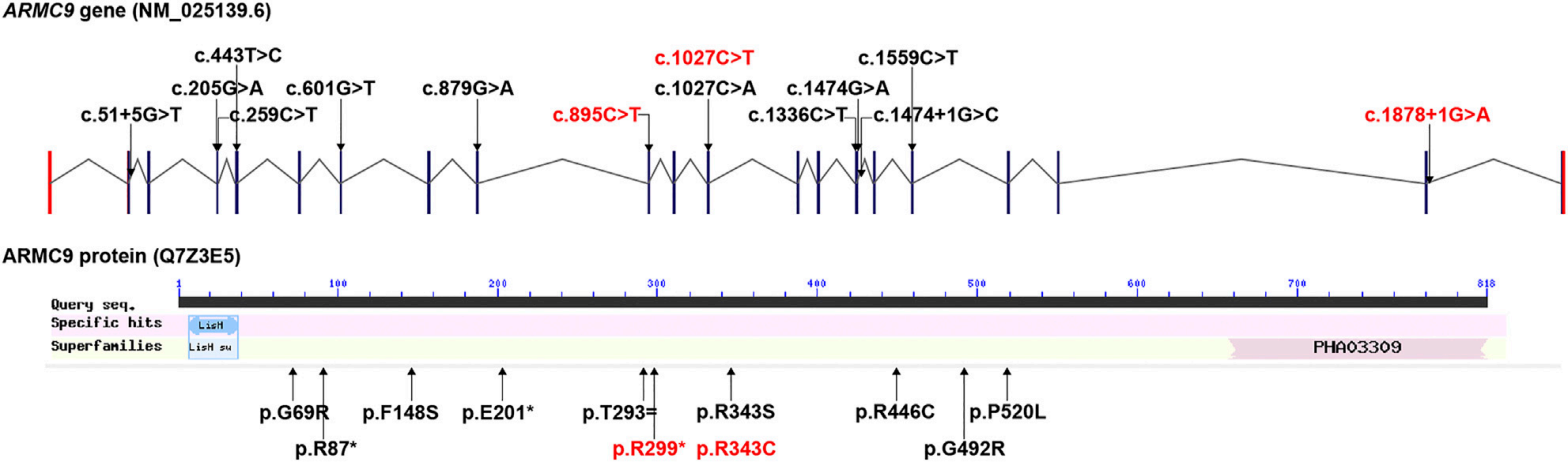
All *ARMC9* variants associated with the JS phenotype reported in the literature, illustrated in gene and protein schematics (red fonts represent the variants in this study).

Collectively, three *ARMC9* variants were detected in this study, namely, a splicing site variant c.1878+1G > A, a nonsense variant c.895C > T, and a missense variant c.1027C > T; out of them, c.1878+1G > A and c.895C > T were newly reported (details in Table1). According to the ACMG guidelines (Richards et al., 2015), both *ARMC9*: c.1878+1G > A and *ARMC9*: c.895C > T (p.Arg299Ter) were predicted to be likely pathogenic mutations because of the evidence chain (PVS1+PM2), and *ARMC9*: c.1027C > T was predicted to be variants of unknown significance (VUS) based on the evidence of PP2, PM2, and PP3. Figure 2 shows the specific locations of the three variants detected in this study in the *ARMC9* gene and protein pattern diagram.

**TABLE 1:** Variants in the *ARMC9* (NM_025139.6) gene associated with the JS phenotype identified in the literature and this study. The amino acid list before is changed to a stop codon (Ter, *)

Patient no	Phenotype	Genomic coordinates	DNA variant	Protein variant	Variation frequencies in gnomAD^a^	HGMD^b^	PMID^c^
1	Developmental delays, tachypnea, apnea, left ptosis, depressed nasal bone, low-set ears	2:232196610	c.1878+1G > A	p.626_627insISATQR	0	—	This study
		2:232121314	c.895C > T	p.Arg299Ter	0.0000119	—	
2	Motor and language delays, mental retardation, abnormal skull and teeth, ptosis, hypotonia, unsteady walking, apnea	2:232196610	c.1878+1G > A	p.626_627insISATQR	0	—	This study, 28625504 Van De Weghe et al. (2017)
		2:232127019	c.1027C > T	p.Arg343Cys	0.0000517	DM	
3	Developmental delays, abnormal eye movements, hysterectomy 2016 (heavy bleeding), worsening visual acuity, seizures	2:232079571	c.205G > A	p.Gly69Arg	0.00001591	DM	28625504 Van De Weghe et al. (2017)
		2:232141350	c.1336C > T	p.Arg446Cys	0.000007958	DM	
4	Developmental delays, abnormal eye movements, polydactyly, ptosis, lithium-induced hypothyroidism	2:232079571	c.205G > A	p.Gly69Arg	0.00001591	DM	28625504 Van De Weghe et al. (2017)
		2:232141350	c.1336C > T	p.Arg446Cys	0.000007958	DM	
5	Developmental delays, abnormal eye movements, retinal dystrophy, abnormal electroretinogram	2:232071007	c.51+5G > T	Splicing	0	DM	28625504 Van De Weghe et al. (2017)
6	Developmental delays, tachypnea, abnormal eye movements, ptosis, seizures	2:232127019	c.1027C > T	p.Arg343Cys	0.00005170	DM	28625504 Van De Weghe et al. (2017)
		2: 232137668	c.1211_1334del	p.Arg405Alafs∗7	—	—	
7	Developmental delays, tachypnea, abnormal eye movements	2:232079625	c.259C > T	p.Arg87∗	0	DM	28625504 Van De Weghe et al. (2017)
		2:232127019	c.1027C > A	p.Arg343Ser	0.000003977	DM	
8	Developmental delays, abnormal eye movements, single heterotopia (left occipital horn)	2:232079625	c.259C > T	p.Arg87∗	0	DM	28625504 Van De Weghe et al. (2017)
		2:232127019	c.1027C > A	p.Arg343Ser	0.000003977	DM	
9	Developmental delays, apnea, tachypnea, abnormal eye movements, ptosis	2:232127019	c.1027C > T	p.Arg343Cys	0.00005170	DM	28625504 Van De Weghe et al. (2017)
10	Developmental delays, apnea, abnormal eye movements, ptosis, micrognathia, high palate, bifid uvula, bilateral optic nerve hypoplasia, GH deficiency, micropenis, eyelid implants, possible hearing loss, borderline HSM	2:232141489	c.1474+1G > C	Splicing	0	DM	28625504 Van De Weghe et al. (2017)
		2:232127019	c.1027C > T	p.Arg343Cys	0.00005170	DM	
11	Developmental delays, tachypnea, ptosis, Dandy Walker malformation, ventriculo- and cysto-peritoneal shunts, non-ambulatory, non-verbal at age 8 years	2:232141488	c.1474G > A	p.Gly492Arg	0.000003984	DM	28625504 Van De Weghe et al. (2017)
		2:232127019	c.1027C > T	p.Arg343Cys	0.00005170	DM	
12	Developmental delays, abnormal eye movements, retinal dystrophy, ptosis, broad nasal bridge; thin upper lip; Y-shaped 2/3 toe syndactyly	2:232146779	c.1559C > T	p.Pro520Leu	0.000003977	DM	28625504 Van De Weghe et al. (2017)
13	Developmental delays, tachypnea, ptosis	2:232146779	c.1559C > T	p.Pro520Leu	0.000003977	DM	28625504 Van De Weghe et al. (2017)
14	Mental retardation, ptosis, polydactyly	2:232104754	c.879G > A	p.Thr293Thr	0.000008065	DM	29159890 Kar et al. (2018)
15	Brain malformations	2:232081445	c.443T > C	p.F148S	0	DM	32304219 Becher et al. (2020)
16	Abnormal profile, cleft palate, encephalocele, holoprosencephaly, polydactyly	2:232091490	c.601G > T	p.Glu201Ter	0	DM	30712878 Petrovski et al. (2019)

a gnomAD exomes (http://gnomad.broadinstitute.org/).

b HGMD®: Human Gene Mutation Database (Professional Version 2019.4); DM: disease causing mutation.

c PMID: PubMed ID(https://pubmed.ncbi.nlm.nih.gov/).

### Intramolecular Impact of the c.1027C > T: p.Arg343Cys Variant

**FIGURE 3:**
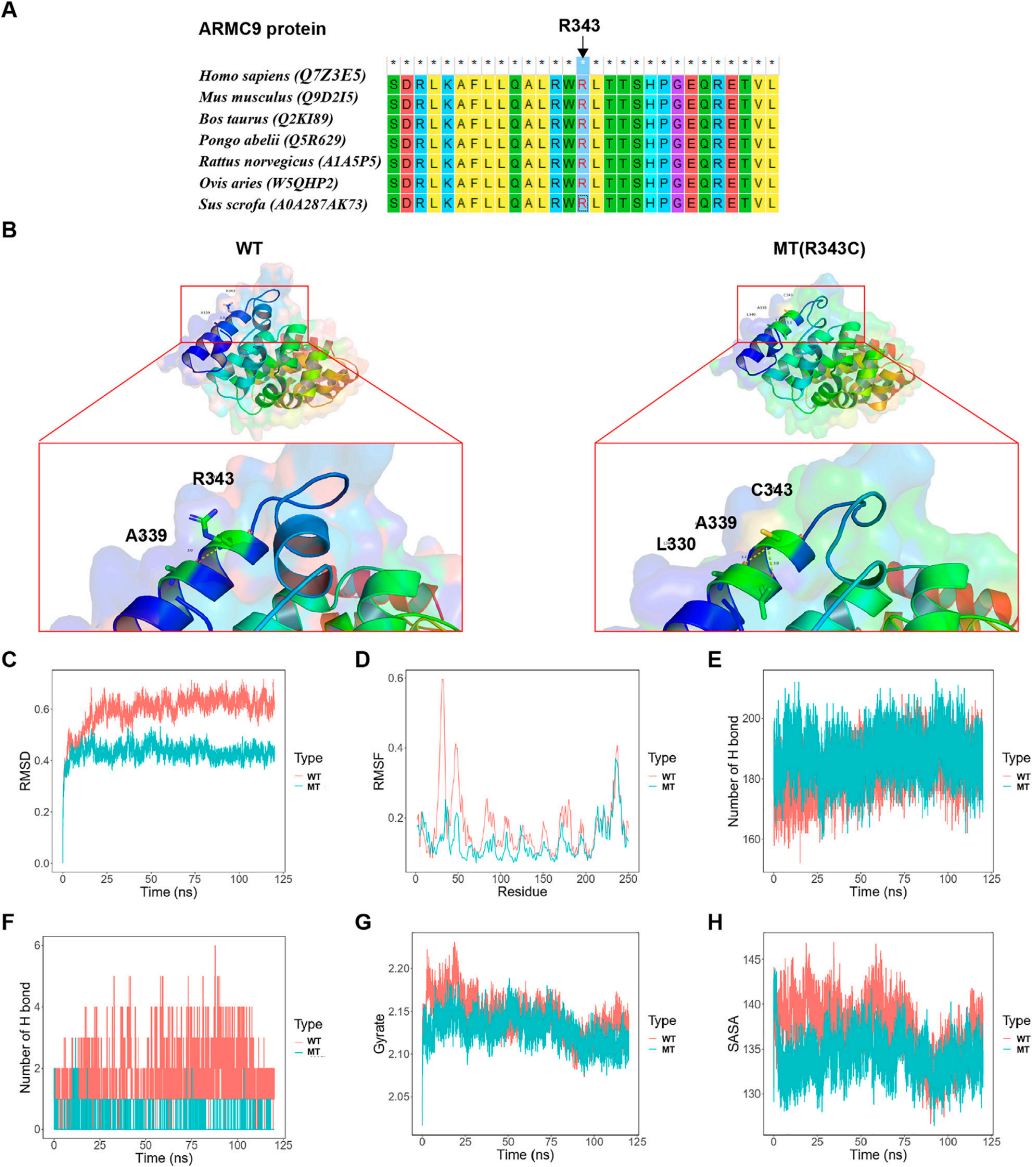
Analysis results on the c.1027C > T: p. Arg343Cys variant. **(A)** The conservation of ARMC9: Arg343 residue across species. **(B)** The structures of the domain containing the WT and Arg343Cys models (residues forming hydrogen bonds with the residue Arg343 or Arg343Cys are depicted in stick representation; dotted yellow lines represent the hydrogen bonds). **(C)** The trajectory of RMSD (Ca) for the two proteins, which compared every structure in the trajectory to the reference/initial frame (0 ns) by computing the root mean square deviation (RMSD). RMSD is a numerical measurement representing the difference between two structures. In molecular dynamics, we are interested in how structures and parts of structures change over time as compared to the starting point, so the trajectory of RMSD can be used to identify large changes in protein structure as compared to the starting point. **(D)** RMSF of the two proteins calculated from each simulation, which computed the root mean square fluctuation (RMSF) of atomic positions in the trajectory after fitting to the reference/initial frame (0 ns). RMSF is a numerical measurement similar to RMSD, but instead of indicating positional differences between entire structures over time, RMSF is a calculation of individual residue flexibility, or how much a particular residue moves (fluctuates) during a simulation. **(E)** The number of hydrogen bonds formed in the wild-type or mutated protein. Although the hydrogen bond is much weaker than a covalent bond, the large number of imide and carbonyl groups in peptide chains results in the formation of numerous hydrogen bonds, and these are important for structures to stabilize the folding of the peptide backbone and facilitate molecular interactions. **(F)** The number of hydrogen bonds formed between the residue Arg343 or Arg343Cys and the other residues for each structure in the trajectory. **(G)** Rg analysis of wild-type ARMC9 and its variants. Rg is a measurement of structural displacement of protein atoms from their common center of mass throughout the simulation and provides comprehensive information on protein compactness over time. **(H)** SASA analysis of wild-type ARMC9 and its variants. SASA measures the exposed surface in protein structures accessible to solvent molecules, and provides relevant information of exposure to its solvent environment over time. WT, wild-type; MT, mutant type with p. Arg343Cys variant.

It was shown that the conservation of Arg343 residue in the ARMC9 protein, which was affected by c.1027C > T: p. Arg343Cys in the proband in family 2, was strictly maintained across species (Figure 3A). The final and converged models out of structure prediction are depicted in Figure 3B.

Based on the MD analysis, it could be inferred from the trajectory of root mean square deviation (RMSD, Figure 3C) and root mean square fluctuation (RMSF, Figure 3D) that the Arg343Cys model was less flexible than the wild-type (WT) model. The mutant model formed a greater number of whole hydrogen bonds than the WT (Figure 3E). Interestingly, the Arg343Cys residue itself formed less hydrogen bonds with the other residues than the WT residue Arg343 (Figure 3F). Furthermore, Arg343Cys increased the compactness of the protein (Figure 3G) and reduced exposed surface in the protein structure (Figure 3H).

### Impact of the c.1878+1G > A Splicing Site Variant

**FIGURE 4:**
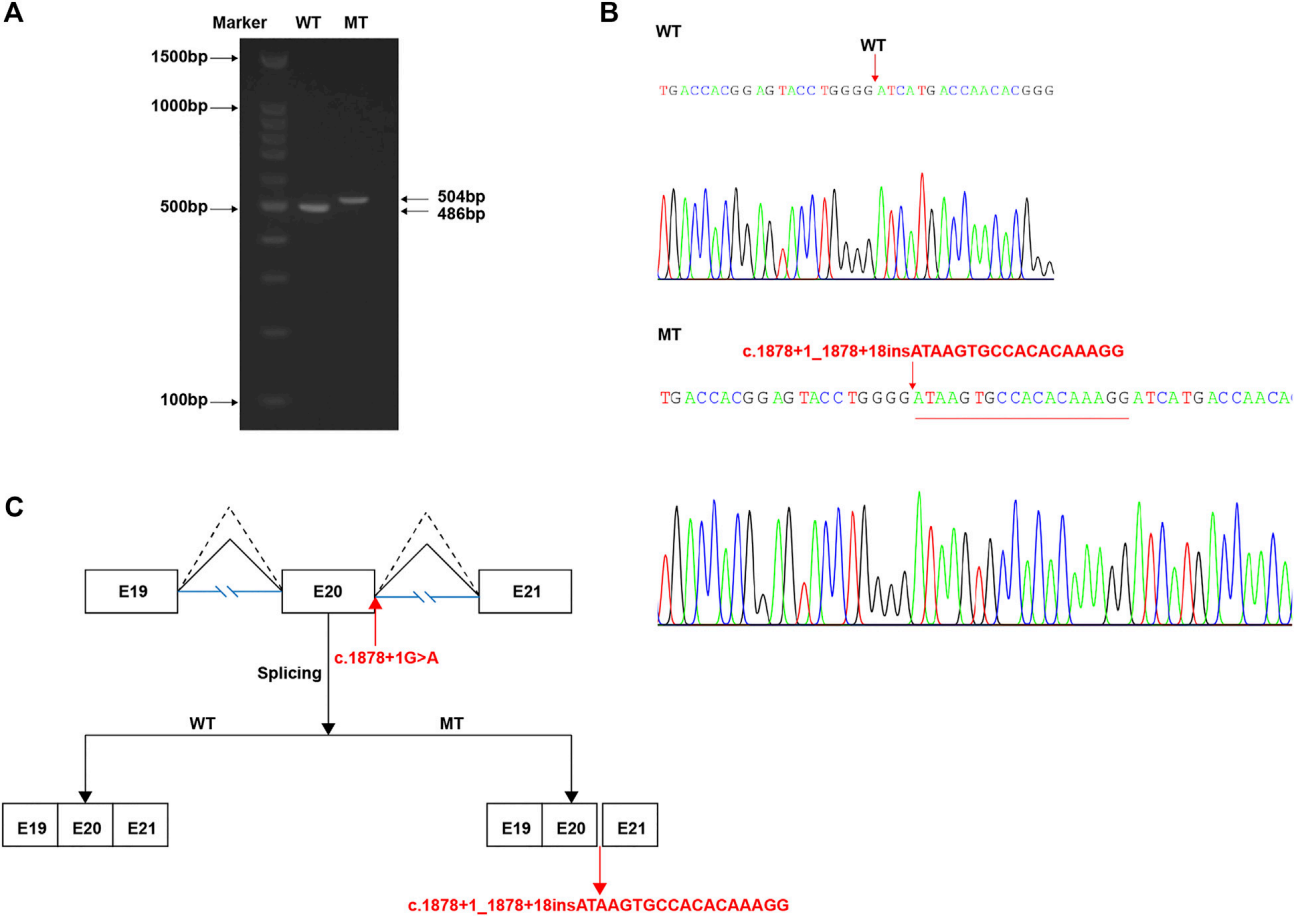
*In vitro* study of the c.1878+1G > A variant. **(A)** After transfection with wild-type (WT) or mutant (MT) vectors, RNA extraction, and reverse transcription, the product results of PCR amplification of target region displayed by agarose gel electrophoresis. **(B)** Sanger sequencing results of cDNA corresponding to the amplification products in **(A)**, indicating a 18 bp insertion. **(C)** The schematic demonstrating the proper (WT, dotted broken lines) and abnormal (MT, full broken lines) splicing patterns by each vector.

We thought the c.1878+1G > A splicing site variant is likely disrupting the canonical splicing donor site (GT). To verify whether this variant causes the splicing machine to look for a new donor site, we constructed the WT and mutant type (MT) with the c.1878+1G > A variant minigene plasmids *in vitro*. According to the *in vitro* experiment, it was demonstrated that the WT plasmid could normally produce the 486 bp transcript as expected (WT lane, Figure 4A), while the MT plasmid produced a longer transcript (MT lane, Figure 4A). Meanwhile, Sanger sequencing of complementary DNA (cDNA) revealed an 18 bp partial intronic retention at the mutant splicing site (Figure 4B, corresponding mRNA splicing diagram shown in Figure 4C). So, the inferred abnormal splicing pattern resulting from the c.1878+1G > A variant was shown in Figure 4C. Correspondingly, it would most likely produce a peptide chain with an insertion, namely, p.626_627insISATQR.

## Discussion

Although Joubert syndrome and related disorders (JSRD) have been generally recognized as a canonical ciliopathy with overlapping characteristics and molecular mechanisms involving cilium dysfunction (Badano et al., 2006; Doherty, 2009), the common cellular dysfunction across genetics of JSRD is still elusive (Bachmann-Gagescu et al., 2015; Parisi, 2019).

Mutations in the *ARMC9* gene were first proven as a cause of JS by Van De Weghe et al. based on human genetic, protein localization, and zebrafish model data in 2017 (Van De Weghe et al., 2017). In their study, patients with *ARMC9* biallelic mutations showed typical JS manifestations, such as “molar tooth sign,” and also some degree of phenotypic variability, mainly in the presence of apnea, ptosis, and polydactyly. After that, Kar reported an Indian familial JS case with *ARMC9* variation, which further supported this etiology. Petrovski et al*.* reported a patient with homozygous nonsense *ARMC9* mutation presenting abnormal profile, cleft palate, encephalocele, holoprosencephaly, and polydactyly (Petrovski et al., 2019). Becher et al. reported a patient with homozygous missense *ARMC9* mutation with just “brain abnormalities” but no specific information (Becher et al., 2020). In Table 1, we summarize all the *ARMC9* variants reported so far which are associated with JSRD or similar phenotypes (Van De Weghe et al., 2017; Kar et al., 2018; Petrovski et al., 2019; Becher et al., 2020). In our study, both patients presented typical “molar tooth sign” by MRI, but did not display polydactyly, renal, or hepatic phenotype; the breath pattern of Patient two was relatively normal. We speculated that the phenotypic variation should be related to the types and locations of specific *ARMC9* mutations. However, due to the small amount of patients and mutations discovered at present, it is not possible to establish the rule.

Herein, we analyzed two variants, namely, *ARMC*: c.1027C > T: p. Arg343Cys (missense) and c.1878+1G > A (splicing site), by *in silico* and minigene construction, respectively. In regard to the former, the conservation of the affected residue (Arg343) maintained across species, which indirectly supported this variant being deleterious. Moreover, located at the outer helix of the protein, this variant would probably alter the state of hydrogen bonds formed between this residue and others, which in turn may affect the protein–protein interaction. Besides, MD results obviously indicated that the p. Arg343Cys variant would most likely affect the corresponding secondary structure of the ARMC9 protein. However, these *in silico* predictions still need to be backed up by functional experiments. In regard to the c.1878+1G > A variant, the *in vitro* experiment showed that it can introduce an 18 bp insertion to the coding sequence, which most likely results in a 6-amino acid redundancy. It will be interesting to investigate how this abnormal protein changes its protein function and affects the development of primary cilia. In addition, both of our patients carried this variant, suggesting that it may be recurrent and should be of concern in the Chinese population. Yet this also needs to be supported by data from a larger sample size.

At present, only a few studies on the function of the ARMC9 protein emerged. Van De Weghe et al*.* provided evidence that ARMC9 localizes to the ciliary basal body, which originates from the mother centriole that docks at the cell membrane during interphase to nucleate the ciliary axoneme; ARMC9 is present in ciliated organisms and upregulated in ciliated cells, and zebrafish harboring armc9 mutations display typical ciliopathy phenotypes (Van De Weghe et al., 2017). Louka et al. revealed ARMC9 as a new distal segment boundary protein that localizes near the ends of B-tubules in the cilia and acts as a negative regulator of B-tubule length in *Tetrahymena* model, and loss of ARMC9 could affect parameters of cilia (number, length, and beat parameters) and reduce cilia-dependent functions (Louka et al., 2018). Latour et al. identified the coiled-coil domain containing 66 (CCDC66) and TOG array regulator of axonemal microtubules 1 (TOGARAM1) as an ARMC9 interaction partner and demonstrated that dysfunction of ARMC9 or TOGARAM1 resulted in short cilia with decreased axonemal acetylation and polyglutamylation, but relatively intact transition zone function in zebrafish (Latour et al., 2020). In general, further exploration of the *ARMC9* gene at both clinical and functional levels will help us to understand the pathogenesis of JS.

In conclusion, the current study established the genetic diagnosis on 2 JS cases *via* WES detection, and identified 2 novel *ARMC9* variants, which expanded its mutation spectrum. The following *in silico* and *in vitro* studies strongly supported the pathogenicity of these variants. Our findings provide a solid evidential basis for genetic counseling to the affected families and shed light on the importance of ARMC9 function in JS pathogenesis.

## Data Availability

The datasets for this article are not publicly available due to concerns regarding participant/patient anonymity. Requests to access the datasets should be directed to the corresponding author.
