# Supplementary material for: Whole Exome Sequencing Identified Novel ARMC9 Variations in Two Cases With Joubert Syndrome
Source: Front Genet. 2022 Feb 4;13:817153. doi: 10.3389/fgene.2022.817153 (PMC8855066; doi:10.3389/fgene.2022.817153)
Supplement: Supplementary file 1 [file Table2.docx]

**Supplementary material 1.**

**Table. PCR Primers for Sanger sequencing validation**

| **Primers** | **Sequence** | **Length** | **Tm** |
| --- | --- | --- | --- |
| *ARMC9*-exon10-F | CCGTGCATTGCTATCAGTGTT | 219 | 60℃ |
| *ARMC9*-exon10-R | CATGTAGCTCTAGCTCAAAGGCA |  |  |
| *ARMC9*-exon12-F | TCTCGTTTTGGTTGGCTGGT | 310 | 60℃ |
| *ARMC9*-exon12-R | AGAATTGAAGGCCCTGGCTG |  |  |
| *ARMC9*-exon20-F | TGCAAGTTGCTGTGATTGGC | 403 | 60℃ |
| *ARMC9*-exon20-R | TAGGGCCACAGTGCATTTCA |  |  |
