# Supplementary material for: Whole Exome Sequencing Identified Novel ARMC9 Variations in Two Cases With Joubert Syndrome
Source: Front Genet. 2022 Feb 4;13:817153. doi: 10.3389/fgene.2022.817153 (PMC8855066; doi:10.3389/fgene.2022.817153)
Supplement: Supplementary file 2 [file DataSheet1.docx]

**Supplementary material 2.**

Detailed methods of the *in vitro* experimental validation.

**1. Diagram of the backbone of pMini-CopGF expression plasmid.**


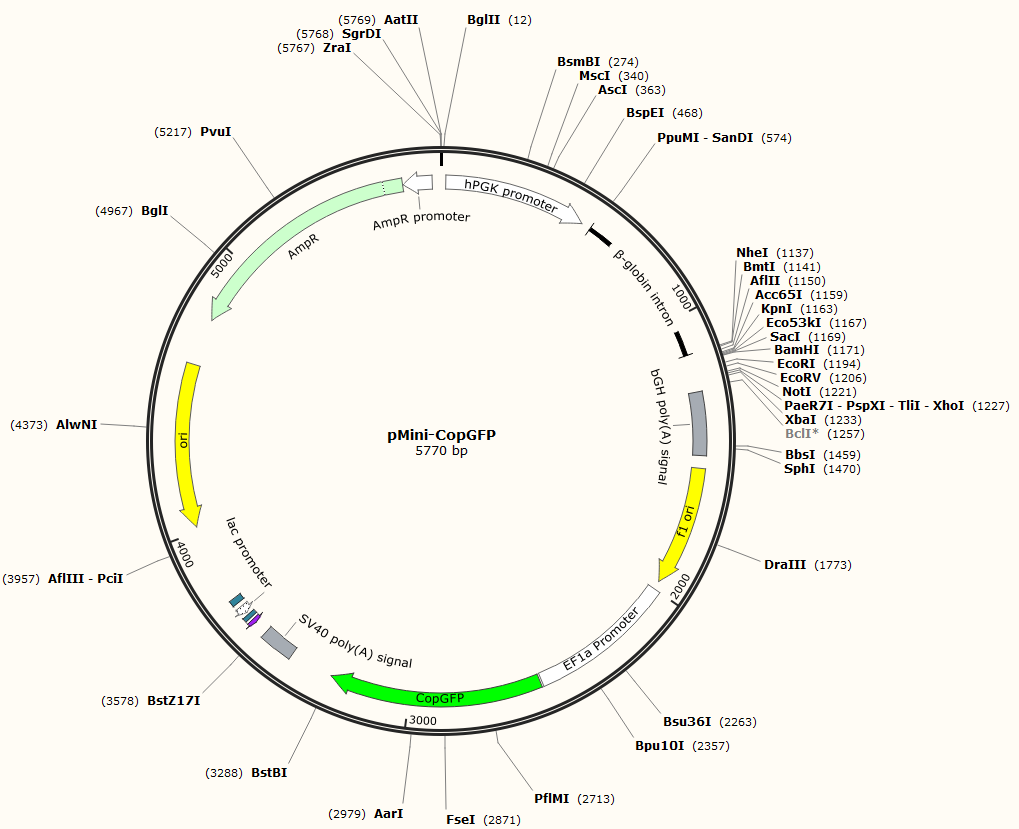


**2. Restriction enzyme cutting sites:**

BamHI/XhoI

**3. Inserted sequences**

Note: The inserted sequence were designed to contain 3 exons (E19, E20 and E21) and 2 introns (Ivs19 and Ivs20). As intron19 and intron20 are too large, both introns adopt a resolution strategy (the middle sequence of introns is deleted, and the sequences at the front and rear ends are retained for recombination). The whole sequences are as follows (red letters, exonic sequence; black letter, intronic sequence; letters magnified on a yellow background represent the mutation site):

3.1 ARMC9-WT (NM_025139.6)

aagagctaccagatggtgttcttgaatctgatgatgatgaagatgaagatgatgaagtaagttggaggttcttgacaccacatagaaaacccataccaaactattctctctttcctgccttgacaagttagtccagaaatggagatttttcattcctgctacactatttctcacaattctgagggttggctaaagctcagctgggtggtggttcgggtctcacttggggtctcatgtggggagcccagcggccatgtttgaagactcccacactcattgggttcacagcagagacgcagcattgcatgtcattcgagtttcatgggaagctgaacaatgaccattcttctgtgtgagcttttgctctgaaggggcacgagcctcggcctgtcttcctcaccggtgggagtgctgggcgtgtaggcgcctggcgagagtctctggcgtgaggggtcctgggtttagttcagagctaacggaactgaccagttttctctgttatttgcattcttttgacttgtgtttcctcagctgtgaaaaatctacttgcctggtgggaatatggaaaggaacatccactgggtcttcccagaagcagaacggtgctgccctttttgctgagtcttgttacttattggctgtaaacgttcttgagatgagcagacctgttctagagttccctatcccccataaggcctgtgtatgactgactggctgttctatatggtgccttgatctcacagagagagaagttctgagaactgtgattaggagagactttaaagagccttctggaacctccgcacaactaagcagtggaggctccctagctccctagcttcctgaagaggctggttggccttgtccctttggtcttcatggagaggttgggagcagaggcagactgcccctgagcccccttggaatataagcctgcaagttgctgtgattggccgaggccttcttgtttcatgacagctacacattctggggagccacgccttgggagcttgtgtcagggtgtccatggcattcaccccatgtctcctgaaacaggaggaccatgacatcatggaagccgatctggacaaagacgaactgatccagccccagctcggagaactctcaggagagaagcttctgaccacggagtacctgggggtaagtgccacacaaagggtggggatcctgaaacagaaaggccagatggacattgatggatttcgtgttttcagtgacacagagggtttagtttggtttgttaccatactgtatttatgagaaggtccatgttagccagtctgctctcagtggccctgaaatgcactgtggccctagctacctgtcacaagtagacaggtgtacggattggggaaagatataactagaaggcatatgccaggggcctttggaacatcagaagatatgcctggccccagtagcaattgcctaccatcccactttttaatttctgcatgccttatgtattggaagggaatactgaggtagacttggaagtccttggcgcagaggtgttggctgaaaccatcatagcccatgaggttacccactgaagcacagtgtggagagagagtgggggctgagcaaggaggggcattccagcagtcaggagtgggagaaaggagaggacccgagaaggccagaaagtgagtagtcactgcgtcttcaggagaactgaagagggttatggaaacagaagggagaggtgtttcaggatggagccagtggtcagtgcaagcatccaagaggcctgtggagtcaccatctgggatgggtccttaagctgacctgtgagagctcccgttggctcggatgctcagccagctccagaaccactggcttacgacccaccagctaaatagggcaggatgtgagtcaggaggaagcagccactcctcaggagaggatagcagtgccagggcccgcagatatctcagagagcaggtaggggggcctggtgggatgctggagtgtaagggtctggaagggaggagcaattgccaggaaccaggaccaattcagactgaaaccctgtgtgggacagctgagcacatagggtagaagagtcactaaaagaggctcctagaactttgaagctggtggttgcttcagtcagttgtttggaccccaaagtcttccttgttttacttactgatttgtaggagctccttagctatgaaaatgttaacctttaatctgttaaatttgcttcaaatacttcttccaagtttgttgtttgcctgttgatttggtttatttttaacatactgcgcatgtggaaatgttcgtatttttatgaaatcaaatgtccatctttttttcccttctcatttttacaaacccagtccttttcagagatgtgatgagttccagtagatcaaccgctttcttccttttcctagggtttgactttgttttaatttatactgctaaatggtgtgatacacaaaccgttagcaaaaaaagggtttttccccacaatttctgtttacctgaataatttgtgacatgatccttcctcatttatttgcaaagcttcttatcattatagagtagtttattcagtgtattctgcttgacttctaggtcagctctctaatagatatttctccagcccttttttctctttccttccaccagatcatgaccaacacggggaagacaaggcggaaggggctggctaatgtgcagtggagcggggatgagcccctgcaaaggcccgtcacccccggcggccacagaaacgggtacccagtgtaagtcagggctaaaggaagcgggaattgactttcttaagctttgttttgattacagtgtaagatgtatgtatttttaaaattcaaaataaagcattcattttgaaaagca

3.2 ARMC9-MT (NM_025139.6: c.1878+1G>A)

aagagctaccagatggtgttcttgaatctgatgatgatgaagatgaagatgatgaagtaagttggaggttcttgacaccacatagaaaacccataccaaactattctctctttcctgccttgacaagttagtccagaaatggagatttttcattcctgctacactatttctcacaattctgagggttggctaaagctcagctgggtggtggttcgggtctcacttggggtctcatgtggggagcccagcggccatgtttgaagactcccacactcattgggttcacagcagagacgcagcattgcatgtcattcgagtttcatgggaagctgaacaatgaccattcttctgtgtgagcttttgctctgaaggggcacgagcctcggcctgtcttcctcaccggtgggagtgctgggcgtgtaggcgcctggcgagagtctctggcgtgaggggtcctgggtttagttcagagctaacggaactgaccagttttctctgttatttgcattcttttgacttgtgtttcctcagctgtgaaaaatctacttgcctggtgggaatatggaaaggaacatccactgggtcttcccagaagcagaacggtgctgccctttttgctgagtcttgttacttattggctgtaaacgttcttgagatgagcagacctgttctagagttccctatcccccataaggcctgtgtatgactgactggctgttctatatggtgccttgatctcacagagagagaagttctgagaactgtgattaggagagactttaaagagccttctggaacctccgcacaactaagcagtggaggctccctagctccctagcttcctgaagaggctggttggccttgtccctttggtcttcatggagaggttgggagcagaggcagactgcccctgagcccccttggaatataagcctgcaagttgctgtgattggccgaggccttcttgtttcatgacagctacacattctggggagccacgccttgggagcttgtgtcagggtgtccatggcattcaccccatgtctcctgaaacaggaggaccatgacatcatggaagccgatctggacaaagacgaactgatccagccccagctcggagaactctcaggagagaagcttctgaccacggagtacctggggataagtgccacacaaagggtggggatcctgaaacagaaaggccagatggacattgatggatttcgtgttttcagtgacacagagggtttagtttggtttgttaccatactgtatttatgagaaggtccatgttagccagtctgctctcagtggccctgaaatgcactgtggccctagctacctgtcacaagtagacaggtgtacggattggggaaagatataactagaaggcatatgccaggggcctttggaacatcagaagatatgcctggccccagtagcaattgcctaccatcccactttttaatttctgcatgccttatgtattggaagggaatactgaggtagacttggaagtccttggcgcagaggtgttggctgaaaccatcatagcccatgaggttacccactgaagcacagtgtggagagagagtgggggctgagcaaggaggggcattccagcagtcaggagtgggagaaaggagaggacccgagaaggccagaaagtgagtagtcactgcgtcttcaggagaactgaagagggttatggaaacagaagggagaggtgtttcaggatggagccagtggtcagtgcaagcatccaagaggcctgtggagtcaccatctgggatgggtccttaagctgacctgtgagagctcccgttggctcggatgctcagccagctccagaaccactggcttacgacccaccagctaaatagggcaggatgtgagtcaggaggaagcagccactcctcaggagaggatagcagtgccagggcccgcagatatctcagagagcaggtaggggggcctggtgggatgctggagtgtaagggtctggaagggaggagcaattgccaggaaccaggaccaattcagactgaaaccctgtgtgggacagctgagcacatagggtagaagagtcactaaaagaggctcctagaactttgaagctggtggttgcttcagtcagttgtttggaccccaaagtcttccttgttttacttactgatttgtaggagctccttagctatgaaaatgttaacctttaatctgttaaatttgcttcaaatacttcttccaagtttgttgtttgcctgttgatttggtttatttttaacatactgcgcatgtggaaatgttcgtatttttatgaaatcaaatgtccatctttttttcccttctcatttttacaaacccagtccttttcagagatgtgatgagttccagtagatcaaccgctttcttccttttcctagggtttgactttgttttaatttatactgctaaatggtgtgatacacaaaccgttagcaaaaaaagggtttttccccacaatttctgtttacctgaataatttgtgacatgatccttcctcatttatttgcaaagcttcttatcattatagagtagtttattcagtgtattctgcttgacttctaggtcagctctctaatagatatttctccagcccttttttctctttccttccaccagatcatgaccaacacggggaagacaaggcggaaggggctggctaatgtgcagtggagcggggatgagcccctgcaaaggcccgtcacccccggcggccacagaaacgggtacccagtgtaagtcagggctaaaggaagcgggaattgactttcttaagctttgttttgattacagtgtaagatgtatgtatttttaaaattcaaaataaagcattcattttgaaaagca

**4. Primer sequences used for RT-PCR amplification and cDNA Sanger sequencing**

| MiniRT-F | GGCTAACTAGAGAACCCACTGCTTA |
| --- | --- |
| MiniRT-R | GTTTAAACGGGCCCTCTAGACTCGA |

**5. Normal transcriptional result sequence as expected** (size 486 kb; black font is the vector transcription sequence, and the red font is the exon transcription sequence of the target gene)

GGCTAACTAGAGAACCCACTGCTTACTGGCTGCTAGCGTTTAAACTTAAGCTTGGTACCGAGCTCGGATCCaagagctaccagatggtgttcttgaatctgatgatgatgaagatgaagatgatgaagaggaccatgacatcatggaagccgatctggacaaagacgaactgatccagccccagctcggagaactctcaggagagaagcttctgaccacggagtacctggggatcatgaccaacacggggaagacaaggcggaaggggctggctaatgtgcagtggagcggggatgagcccctgcaaaggcccgtcacccccggcggccacagaaacgggtacccagtgtaagtcagggctaaaggaagcgggaattgactttcttaagctttgttttgattacagtgtaagatgtatgtatttttaaaattcaaaataaagcattcattttgaaaagcaCTCGAGTCTAGAGGGCCCGTTTAAAC
